# The Effect of Oral Dexmedetomidine Premedication on Preoperative Cooperation and Emergence Delirium in Children Undergoing Dental Procedures

**DOI:** 10.1155/2017/6742183

**Published:** 2017-08-20

**Authors:** Sultan Keles, Ozlem Kocaturk

**Affiliations:** ^1^Department of Pediatric Dentistry, Faculty of Dentistry, Adnan Menderes University, Aydın, Turkey; ^2^Department of Oral and Maxillofacial Surgery, Faculty of Dentistry, Adnan Menderes University, Aydın, Turkey

## Abstract

**Introduction:**

The aim of this study was to detect the effect of 1 *μ*g/kg of oral dexmedetomidine (DEX) as premedication among children undergoing dental procedures.

**Materials and Methods:**

The study involved 100 children between 2 and 6 years of age, ASA I, who underwent full-mouth dental rehabilitation. The DEX group (*n* = 50) received 1 *μ*g/kg DEX in apple juice, and the control group (*n* = 50) received only apple juice. The patients' scores on the Ramsay Sedation Scale (RSS), parental separation anxiety scale, mask acceptance scale, and pediatric anesthesia emergence delirium scale (PAEDS) and hemodynamic parameters were recorded. The data were analyzed using chi-square test, Fisher's exact test, Student's *t*-test, and analysis of variance in SPSS.

**Results:**

RSS scores were significantly higher in the DEX group than group C at 15, 30, and 45 min (*p* < 0.05). More children (68% easy separation, 74% satisfactory mask acceptance) in the DEX group showed satisfactory ease of parental separation and mask acceptance behavior (*p* < 0.05). There was no significant difference in the PAEDS scores and mean hemodynamic parameters of both groups.

**Conclusions:**

Oral DEX administered at 1 *μ*g/kg provided satisfactory sedation levels, ease of parental separation, and mask acceptance in children but was not effective in preventing emergence delirium. The trial was registered (Protocol Registration Receipt NCT03174678) at clinicaltrials.gov.

## 1. Introduction

General anesthesia is frequently used in the treatment of dental caries in early childhood [1, 2]. The most recognized indications of general anesthesia are children who cannot cooperate because of a lack of psychological or emotional maturity or mental, physical, or medical disability; those for whom local anesthesia is ineffective because of acute infection, anatomic variations, or allergy; those who are extremely uncooperative, fearful, anxious, and uncommunicative; those requiring extensive surgical procedures; and those requiring immediate comprehensive dental care [2].

Children undergoing operation under general anesthesia may experience preoperative anxiety and may be uncooperative [3]. Uncooperative behaviors of children may be observed at the time of separation from parents, venipuncture, or mask application. Untreated anxiety may lead to difficult induction, increased postoperative pain, greater rescue analgesic needs, emergence delirium (ED), and postoperative psychological effects and behavioral issues [4–6].

There are various agents used in the pediatric population to manage the abovementioned uncooperative behaviors, like midazolam and ketamine. Midazolam has benefits such as anticonvulsant activity, a rapid onset of action, a short duration of action, and a lower incidence of postoperative vomiting. On the other hand, its use is associated with disadvantages such as restlessness, paradoxical reactions, cognitive impairment, postoperative behavioral changes, and respiratory depression [7, 8]. Ketamine has both sedative and analgesic properties. However, adverse effects of ketamine include excessive salivation, nausea and vomiting, nystagmus, hallucination, and postoperative psychological disturbances [9, 10].

Dexmedetomidine (DEX), which is an *α*_2_-adrenoceptor agonist, has been used as a premedicant and analgesic in the pediatric population recently. Several previous studies have evaluated the effect of DEX as a premedicant for various surgeries. However, there is a paucity of studies evaluating the effect of DEX on preoperative anxiety and emergence delirium (ED) in dental restoration surgery [12, 18]. For this reason, the present study was designed with the aim of evaluating the effect of 1 *μ*g/kg oral DEX on preoperative cooperation and emergence delirium in children aged 2–6 years who underwent dental procedures under general anesthesia.

## 2. Materials and Methods

This retrospective study was approved by the Research Ethics Committee of the Adnan Menderes University Faculty of Medicine (2017/1080). Data was collected from patients' files and the study included the children who underwent full-mouth dental rehabilitation between December 2015 and July 2016 and were enrolled in the study.

The inclusion criteria were age between 2 and 6 years and classification as American Society of Anesthesiologists (ASA) grade 1. The exclusion criteria included congenital disease, DEX and propofol allergy, asthma, and mental retardation and those whose parents did not consent to their participation in the study.

Group DEX (*n* = 50) included patients who received 1 *μ*g/kg of oral DEX in apple juice 45 minutes before the induction of anesthesia, whereas group C (the control group, *n* = 50) received plain apple juice orally 45 minutes before the induction of anesthesia.

### 2.1. Study Tools

The following tools were used in this study: the Ramsay Sedation Scale (RSS), parental separation anxiety scale (PSAS), mask acceptance scale (MAS), and the pediatric anesthesia emergence delirium scale (PAEDS). The details of the study evaluation tools and their categorization used in the study are shown in Table 1.

The children in the DEX group were observed by a blinded observer (OK) to determine their levels of sedation per the RSS before premedication and 15 minutes, 30 minutes, and 45 minutes after premedication [11]. The same measurements made in the DEX group were conducted in group C 45 minutes before the operation.

The hemodynamic parameters, heart rate (HR), respiratory rate (RR), and SpO_2_, were assessed before premedication and at 15, 30, and 45 minutes after premedication.

### 2.2. Study Procedures

The study drug was mixed with apple juice (total volume should not exceeded 5 ml) by a nurse and administered by another nurse 45 minutes prior to being transferred to the operating room. Children in control group received only 5 ml of apple juice. The HR, RR, and SpO_2_ values were recorded at baseline and 15, 30, and 45 minutes after the premedication until the patient was transferred to the operating room. A research team member (SK) evaluated the ease of separation of the patients from their parents using the PSAS, approximately 45 minutes after premedication. Another member of the research team calculated the MAS score of each patient. The PAEDS score of each patient was recorded upon discharge from the PACU.

### 2.3. Anesthesia Protocol

All patients were intubated by the same anesthetist. Induction was carried out via a facemask with 8% sevoflurane in 100% oxygen. Following loss of consciousness, an intravenous line was established through which 2 mg/kg of 1% propofol (Propofol-Lipuro®, B. Braun Melsungen AG, Germany), 1 *μ*g/kg of fentanyl (Talinat®, Vem, İstanbul, Turkey), and 0.5 mg/kg of rocuronium (Myocron®, Vem, Istanbul, Turkey) were administered, followed by nasotracheal intubation. The HR, noninvasive blood pressure, and SpO_2_ were monitored for each patient.

Anesthesia was maintained with 2% sevoflurane in a mixture of 50% oxygen and nitrous oxide. All the children received 0.4 mg/kg of tenoxicam (Tilcotil®, Deva, Istanbul, Turkey) for analgesia 15 min before the end of the surgery. The patients were extubated and transferred to the PACU. The patients were observed by a research team member during the entire period of their stay in the PACU, and a PAEDS score was recorded when the patient was fully aroused.

### 2.4. Dental Treatment Procedure

All the patients were treated by the same pediatric dentist (SK). Carious teeth of the patients dental treatment procedures included restorative treatment (compomer, composite, stainless steel crown, pulpotomy, and pulpectomy) or extraction if the tooth was unrestorable.

All treatments were completed in the same general anesthesia session. The durations of the dental operations were recorded in the patients' files.

### 2.5. Statistical Analyses

Based on a previous study [19], minimum required sample size is 49 patients in each group to detect 24% (from 32% to 8%) difference in emergence delirium between DEX and control group at the 0.05 level of significance and to provide 80% power to the study and we rounded the groups up to 50 patients.

The results were presented as the mean ± standard deviation (SD) for quantitative variables and were summarized as absolute frequencies and percentages for categorical variables. Categorical variables were compared using chi-square test or Fisher's exact test if more than 20% of cells with an expected count of less than five were observed. Quantitative variables were also compared with the Student *t*-test. The variations in hemodynamic variables including HR, RR, and SpO_2_, from the baseline among the groups, were analyzed by repeated measures ANOVA.

Statistical analysis was carried out using the SPSS software version 20 (IBM Corp., Armonk, NY, USA). *p* values of 0.05 or less were considered statistically significant.

## 3. Results

There were no statistically significant differences between the groups regarding demographics, duration of operation, and duration of anesthesia (*p* > 0.05). The mean age of the patients was 4.2 ± 1.2 years. The demographic data of the patients are shown in Table 2.

The baseline Ramsay Sedation Scale (RSS) score was comparable in both groups (*p* > 0.05). The value of the RSS score was significantly higher in the DEX group than group C at 15, 30, and 45 min (*p* < 0.05). There was no patient with an RSS score higher than 2. Analysis of the PSAS scores demonstrated that a higher percentage (68%) of the children in the DEX group showed satisfactory response during parental separation (*p* = 0.04).

The analysis in terms of mask acceptance behavior revealed that the DEX group performed better in terms of mask acceptance behavior than group C. It was determined that 74% of the patients in the DEX group and 38% of the patients in group C had a satisfactory mask acceptance behavior and the difference between them was found to be statistically significant (*p* < 0.05).  Table 3 shows the distribution and comparison of sedation satisfaction percentages of the groups in terms of time.

Overall, we did not observe any clinically significant effects of the study drug on SpO_2_ and no patient had a reduction in SpO_2_ to below 95% during the observation period after premedication. There was no significant differences in the mean HRs, SpO_2_, and RR of both groups at baseline, 15, 30, and 45 minutes (*p* > 0.05). The comparison of the mean HR, RR, and SpO_2_ levels of the groups during the premedication period is shown in Table 4.

**Figure 1:**
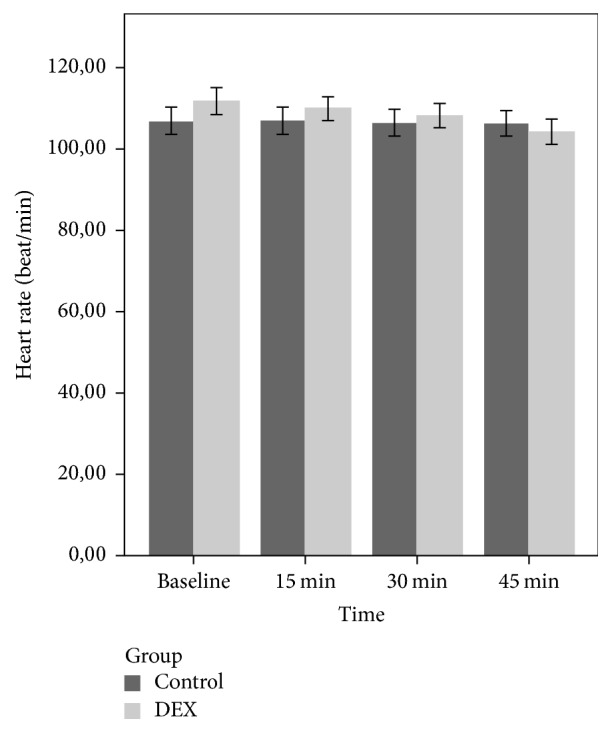
Mean heart rate of the groups during the premedication period.

There were significant group and time effects (*p* < 0.001) and group time interaction on HR (*p* < 0.001) in the DEX group only. The HR reduced significantly from baseline at 15, 30, and 45 minutes after drug administration in the DEX group (*p* < 0.001) (Figure 1).

**Figure 2:**
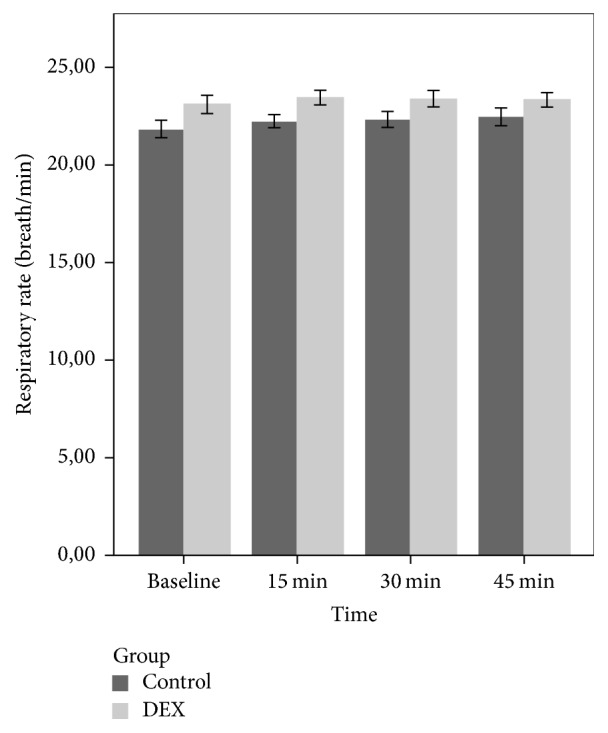
Mean respiratory rate of the groups during the premedication period.

**Figure 3:**
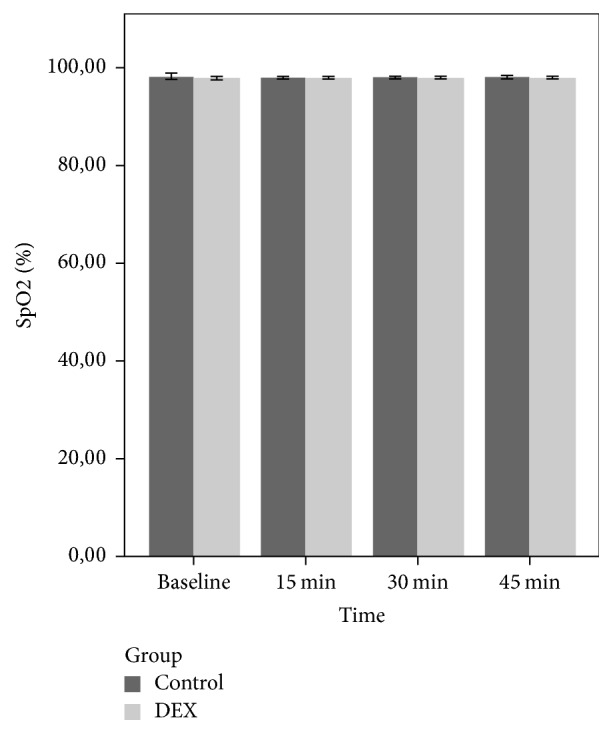
Mean SpO_2_ of the groups during the premedication period.

There was no statistically significant group and time effects and group × time interaction (*p* > 0.05) on RR and SpO_2_ (Figures 2 and 3). In the PACU, children in the DEX group showed lower ED score compared to group C, but the difference was not statistically significant (*p* = 0.11).

## 4. Discussion

This retrospective study determined that 1 *μ*g/kg oral DEX administered as premedication to uncooperative children between the ages of 2 and 6 years who underwent full-mouth dental rehabilitation provided successful sedation at the time of parental separation and mask acceptance during the induction of anesthesia. However, no significant difference was found between both groups in terms of PAEDS scores.

Studies have shown that at least 60% of pediatric patients have preoperative anxiety [3]. The use of sedatives in the preoperative period contributes to the reduction in the patients' level of anxiety, prevents them from experiencing emotional trauma, and induces a calm state during induction of anesthesia.

The majority of children are able to receive dental treatment in a conventional clinical setting. Some patients who are too young or have distinctive fear fail to respond to the usual techniques such as sedation or behavioral management and must, therefore, be treated under general anesthesia [20, 21].

All of the patients included in the present study were young children who were in need of advanced dental treatment and could not be treated with sedation and behavior control techniques. Although the sedatives used in the treatment of preoperative anxiety are quite diverse, DEX has become more frequently used in children [22]. DEX may be administered through the intravenous, oral, buccal, and intramuscular routes with bioavailabilities of 93%, 16%, 82%, and 104%, respectively [23].

Verma et al. compared the acceptability of the nasal and oral routes of administration of midazolam as premedication in children between 2 and 6 years of age and noted that the oral route was considerably more acceptable than the nasal route [24]. For this reason, in this study, the DEX premedication was administered orally, and none of the children rejected taking the premedication.

There are a large number of studies on the administration routes and doses of DEX [18, 25, 26]. However, studies are still underway to determine the optimal route and dosage of DEX. Mountain et al. [12] compared the effect of administering 4 *μ*g/kg of oral DEX and 0.5 mg/kg of midazolam 30 minutes before the operation on mask acceptance behavior and ease of parental separation and ED; they found no statistically significant difference between the groups. Compared to midazolam, which is the gold standard, DEX was found to be sufficient for premedication. However, previous studies noted that DEX might cause cardiovascular complications such as hypotension and bradycardia, depending on the dose and route of administration [25, 27].

Faritus et al. [26] compared the effects of administering 2 *μ*g/kg of oral DEX with 0.5 mg/kg of midazolam as premedication 45 minutes preoperatively in children undergoing congenital heart surgery. A lower dose of DEX than used in previous studies showed similar sedation effects with 0.5 mg/kg of midazolam. Additionally, both agents similarly eased parental separation and mask acceptance behavior, and there were no statistically significant differences between the groups in terms of hemodynamic variables.

Similarly, in the present study, a lower dose than previous studies was used, and satisfactory sedation was achieved in 94% of patients 45 minutes after its administration. However, in 68% of the children, parental separation was eased, while, in 74%, satisfactory mask acceptance was observed. In our study, we considered an RSS score of 2 and above as adequate sedation, meaning the patients were cooperative, awake, oriented, and calm. Thus, in this study, the extent of sedation, mask acceptance, and ease of parental separation were different in comparison with previous studies.

Absorption of a drug through oral mucous membranes is also affected by many factors such as p*K*a, lipophilicity of a drug, and tissue perfusion and uptake. Dexmedetomidine is a highly lipophilic drug with a p*K*a value of 7.1 so that it is mostly in a nonionized form at physiological pH. The nonionized form of dexmedetomidine rapidly crosses biological membranes [28]. The satisfied results obtained from our study with a low dose of dexmedetomidine administered orally for premedication may be achieved due to the fact that children did not swallow the drug immediately and held it in their mouths allowing buccal and/or sublingual absorption.

In previous studies, it has been shown that drugs used for premedication cause changes in vital findings. Kumari et al. compared the effect of 4 *μ*g/kg of oral clonidine, 4 *μ*g/kg of oral DEX, and 0.5 *μ*g/kg of oral midazolam on preoperative cooperation and showed that the mean HR in all groups decreased significantly from the baseline by 30 minutes postoperatively [29]. Yuen et al. found that the HR in children with intranasal administration of DEX as premedication decreased statistically significantly 45 minutes after its administration [25]. In our study, there were no significant differences in the mean HR, RR, and SpO2 values after the administration of DEX.

However, in the repeated measures ANOVA, the HR in the DEX group decreased significantly 15 minutes after drug administration in comparison with group C. Despite this decrease, the values of HR remained within normal hemodynamic limits. The fact that hemodynamic variables remained within normal limits and did not differ from the control group may be because our study used a lower dose of DEX than previous studies.

ED refers to behaviors that include inconsolable crying, thrashing, kicking, disorientation, hallucinations, and cognitive and memory impairment during the recovery period following general anesthesia. These behaviors may result in falling from the bed, increased postsurgical bleeding, and contamination of the surgical wound. Additionally, this situation often requires additional nursing care, additional analgesics, or sedatives which may cause increased hospital stay. In addition, this may cause dissatisfaction of the parents. Although the etiology of ED is not fully known, the intrinsic characteristics of anesthetic agents, rapid emergence from anesthesia, postoperative pain, preschool age, preoperative anxiety, and child temperament are risk factors. Son et al. determined that, in preschool children undergoing anesthesia with sevoflurane without premedication, the incidence of ED was 60% [30]. Özcengiz et al. showed that 2.5 mg/kg of DEX, 0.5 mg/kg of midazolam, and 0.1 mg/kg of melatonin administered orally 45 minutes preoperatively significantly reduced the incidence of ED after anesthesia with sevoflurane [19]. In this study, the overall incidence of ED was 12% and 24% in the DEX group and group C, respectively. Previous studies indicated that the incidence of ED in preschool children varies between 25% and 80% following anesthesia with sevoflurane and desflurane [31, 32].

Sheta et al. reported that the administration of 1 *μ*g/kg of intranasal DEX as premedication resulted in a lower incidence of ED compared to 0.2 mg/kg of intranasal midazolam in children undergoing full-mouth dental rehabilitation [18]. Jannu et al. compared the effect of administering 4 *μ*g/kg of oral DEX as premedication to 0.75 mg/kg of oral midazolam and they observed a lower incidence of ED in children premedicated with DEX [33]. In a similar study, Prabhu and Mehandale reported that 4 *μ*g/kg of oral DEX was superior to oral midazolam when given as premedication in reducing the incidence and severity of ED [34]. Contrary to these studies, the incidence of ED in the DEX group in our study was lower than in group C, although this difference was not statistically significant.

Petroz et al. reported that the terminal half-life of intravenous DEX in children aged between 2 and 12 years was 1.8 hours [35]. The time from the administration of DEX to the measurement of the PAEDS score in our study was longer than the terminal half-life of DEX, suggesting that there was no significant difference between both groups in terms of the incidence of ED.

This clinical study had some limitations. First limitation of this study is its retrospective design. Due to retrospective nature of the study results depended on the records in the patients' files. Second, intravenous formulation of DEX was used as oral preparation of DEX was not available. Third, mixing of the DEX with apple juice could affect pH of the drug and its absorption. Regardless of these limitations there is a strong need for prospective studies with larger sample sizes to find optimum doses of DEX and evaluate its safety and efficacy for pediatric population.

## 5. Conclusions

We concluded that 1 *μ*g/kg of oral DEX provided satisfactory sedation levels and offered significant ease of separation from parents and satisfactory mask acceptance in children. However 1 *μ*g/kg of oral DEX was not effective in preventing emergence delirium.

## Figures and Tables

**Table 1:** Evaluation scales used in the study and their descriptions.

Ramsay Sedation Scale (RSS) [11]	
Score	Description
1	Patient is anxious and agitated or restless or both.
2	Patient is cooperative, oriented, and tranquil.
3	Patient responds to command only.
4	Patient exhibits brisk response to light glabellar tap.
5	Patient exhibits sluggish response to light glabellar tap
6	Patient exhibits no response.

Ramsay sedation score “1” was considered as unsatisfactory and “≥2” was considered as satisfactory sedation.	

Parental separation anxiety scale (PSAS) [12–14]	

Score	Description
1	Child separates easily
2	Child whimpers, but easily assured
3	Crying and cannot or is difficult to be assured
4	Crying and clinging to parents

A score of 1-2 was considered “satisfactory separation” and a score of 3-4 was considered “unsatisfactory separation.”	

Mask acceptance scale (MAS) [14, 15]	

Score	Description
1	Unafraid, cooperative, accepting mask readily
2	Slight fear of mask, easily reassured
3	Moderate fear of mask
4	Terrified, crying, or combative

A score of 1-2 was considered “satisfactory” and a score of 3-4 was considered “unsatisfactory”	

Pediatric emergence delirium scale (PAEDS) [16, 17].	

(1) The child makes eye contact with the caregiver	
(2) The child's actions are purposeful	
(3) The child is aware of his or her surroundings	
(4) The child is restless	
(5) The child is inconsolable	

Items 1, 2, and 3 are reversely scored as follows: 4 = not at all, 3 = just a little, 2 = quite a bit, 1 = very much, and 0 = extremely. Items 4 and 5 are scored as follows: 0 = not at all, 1 = just a little, 2 = quite a bit, 3 = very much, and 4 = extremely. The scores of each item are summed to obtain a total PAEDS score. ED increases directly with the total score. A score ≥10 was considered as presence of emergence delirium.	

**Table 2:** Demographic information, duration of operation, and duration of anesthesia comparison of the groups. Data are expressed as mean ± SD or number of children. Significant differences (*p* < 0.05).

	Group control *n* = 50	Group DEX *n* = 50	*p* value
Age (years)	4.1 ± 1.4	4.3 ± 0.9	0.28
Gender (male/female)	29/21	26/24	0.68
Weight (kg)	16.6 ± 3.2	17.7 ± 4.2	0.16
Duration of operation (min)	58.8 ± 26.1	53.8 ± 25.0	0.33
Duration of anesthesia (min)	76.9 ± 24.8	71.2 ± 25.1	0.25

**Table 3:** Ramsey sedation levels of the groups and comparison of the groups in terms of preoperative cooperation.

Time interval since premedication	Group C *n*/%	Group DEX *n*/%	*x* ^2^/*p* value
*Ramsay baseline*			
Unsatisfactory	25 (50)	25 (50)	0.00/1.00
Satisfactory	25 (50)	25 (50)	

*Ramsay 15 min*			
Unsatisfactory	24 (48)	36 (72)	6.0/0.01^*∗*^
Satisfactory	26 (52)	14 (28)	

*Ramsay 30 min*			
Unsatisfactory	24 (48)	14 (28)	4.24/0.03^*∗*^
Satisfactory	26 (52)	36 (72)	

*Ramsay 45 min*			
Unsatisfactory	24 (48)	3 (6)	22.3/0.00^*∗*^
Satisfactory	26 (52)	47 (94)	

*Successful parental separation*			
Yes	24 (48)	34 (68)	4.1/0.04^*∗*^
No	26 (52)	16 (32)	

*Mask acceptance*			
Satisfactory	19 (38)	37 (74)	13.49/0.00^*∗*^
Unsatisfactory	31 (62)	13 (26)	

*Emergence delirium*			
Present	12 (24)	6 (12)	2.43/0.12
Absent	38 (76)	44 (88)	

Values in number (%). ^*∗*^Significant differences between groups at 0.05 level.

**Table 4:** Comparison of the groups' mean hemodynamic variables in premedication period.

Hemodynamic variables	Group C	Group DEX	*p* value
HR before premedication, beat/min	107 ± 11.4	111.8 ± 11.6	0.85
HR, 15 min after premedication, beat/min	107.5 ± 11.8	110.5 ± 10.4	0.30
HR, 30 min after premedication, beat/min	106.4 ± 11.7	108.3 ± 10.5	0.45
HR, 45 min after premedication, beat/min	106.4 ± 10.8	104.4 ± 10.8	0.79
RR, before premedication, breath/min	21.9 ± 1.4	23.1 ± 1.6	0.32
RR, 15 min after premedication, breath/min	22.2 ± 0.9	23.4 ± 1.2	0.30
RR, 30 min after premedication, breath/min	22.2 ± 1.1	23.4 ± 1.5	0.53
RR, 45 min after premedication, breath/min	22.4 ± 1.1	23.3 ± 1.3	0.10
SpO_2_, before premedication, %	98.0 ± 2.2	97.9 ± 0.8	0.36
SpO_2_, 15 min after premedication, %	98.1 ± 0.8	98.1 ± 0.5	0.58
SpO_2_, 30 min after premedication, %	98.0 ± 0.8	98.1 ± 0.6	0.65
SpO_2_, 45 min after premedication, %	98.1 ± 0.8	98.0 ± 0.7	0.26

Data are presented as mean ± SD.
